# Hemorrhage in Pelvic Ring Fractures After Low-Energy Trauma: A Systematic Review

**DOI:** 10.3390/jcm13237223

**Published:** 2024-11-28

**Authors:** Alina Roßler, Lara Lukhaup, Max Seidelmann, Catharina Gaeth, Sven-Oliver Dietz, Christof Audretsch, Paul Grützner, Joachim Windolf, Anne Neubert

**Affiliations:** 1 Department of Orthopaedics and Trauma Surgery, BG Klinik Ludwigshafen, 67071 Ludwigshafen, Germany; 2 TraumaEvidence @ German Society of Trauma Surgery, 10623 Berlin, Germany; 3 Medical Faculty, University of Heidelberg, 69120 Heidelberg, Germany; 4 Department for Trauma Surgery and Orthopaedics, Reconstructive and Hand Surgery, Burn Medicine, Germany Armed Forces Central Hospital Koblenz, 56072 Koblenz, Germany; 5 Center for Orthopaedics and Trauma Surgery, University Medical Center, Johannes Gutenberg University Mainz, 55131 Mainz, Germany; 6 Department of Trauma and Reconstructive Surgery, Eberhard Karls University of Tuebingen, BG Clinic Tuebingen, 72076 Tübingen, Germany; 7 Department of Orthopedics and Traumatology, University Hospital and Medical Faculty, Heinrich-Heine-University Duesseldorf, 40225 Düsseldorf, Germany

**Keywords:** pelvic fractures, hemorrhage, older adults, systematic reviews, rare complications, fragility fractures, hemorrhagic instability

## Abstract

**Background/Objectives**: The aim was to investigate diagnostic, treatment and preventive options to establish an overview of the existing evidence on hemorrhage in pelvic fractures in older adults. **Methods**: A systematic review was conducted. Due to the rarity of this complication, only case reports and series with individuals older than 55 years with a pelvic ring fracture that is caused by a low-energy trauma or no apparent trauma, along with hemorrhage, were eligible. A search was performed on four databases. The CARE checklist was used to investigate the reporting integrity of the included studies. Analysis was performed narratively, and this study was registered on the Open Science Framework. **Results**: 21 patients from 19 studies were included (17 females and 4 males) with an average age of 82.1 years. The 21 patients suffered a total of 29 fractures. Pubic ramus fractures were present in 48.3% of all fractures. In 42.9%, an active hemorrhage was reported. Arterial vessels were injured in direct anatomical relationship to the fracture. Abdominal pain and hemorrhagic instability were the main red flag symptoms reported. Active arterial hemorrhage was diagnosed by CT with angiogram and treated by embolization with or without additional surgery. **Conclusions**: Clinically relevant hemorrhage in pelvic fractures due to low-energy trauma is rare. However, these fractures, combined with clinically relevant hemorrhage, account for an increased mortality and morbidity in elderly people. This systematic review was able to create a clinical decision tree for hemorrhage in ramus pubic fractures.

## 1. Introduction

Pelvic ring fractures account for about 0.3–8% of all fractures [1]. Females are more often affected than males (70%:30%, respectively) [2]. In young patients, these fractures usually occur as a result of a high-energy trauma like traffic accidents or due to a fall from great heights. In comparison, pelvic ring fractures in older adults usually occur as a result of a low-energy trauma or even without apparent trauma [1,3]. Pelvic injuries in combination with a hemorrhage are potentially life-threatening [4]. The increased use of anticoagulants among older adults further elevates hemorrhage risk [1].

The incidence of pelvic ring fractures in patients older than 85 years is 450 cases per 100,000 population, compared to an incidence of 37 cases per 100,000 population in the general population [1,5]. Specifically, fragility fractures are increasingly common in the older population. This population group is rising as a result of the demographic change, especially in high-income countries [1,3,6,7].

In cases of pelvic ring fractures due to high-energy trauma, highly standardized procedures for diagnostic and therapeutic management are defined. However, the diagnosis of fragility fractures is often delayed, either because patients do not visit the doctor immediately or the correct radiographic diagnostic exams are not performed as a first-line diagnostic measure. Particularly in cases of hemorrhage in patients with fragility fractures of the pelvis (FFPs), an immediate diagnosis of the underlying injury is crucial to reduce mortality and morbidity [1].

So far, little is known about the diagnostics, management and prevention of hemorrhages in FFPs, as these cases are rare. However, with a progressive demographic change, such rare cases are likely to become more common. Hence, there is a need to investigate possible diagnostic, treatment and preventive options to establish an overview of the existing evidence from which guiding principles for this life-threatening condition can be drawn. The aim of this systematic review was to map the existing evidence of diagnostic, treatment and preventive options for hemorrhage in pelvic ring fractures in older adults.

## 2. Materials and Methods

This systematic review was registered on the Open Science Framework (https://doi.org/10.17605/OSF.IO/Y2EBM). It is reported according to PRISMA guidelines [8].

### 2.1. Inclusion Criteria

All inclusion criteria are listed in Table 1. Individuals older than 55 years with a pelvic ring fracture (either pelvis or sacrum fractures), diagnosed via X-ray, computer tomography (CT) scan or magnetic resonance imaging (MRI), that are caused by a low-energy trauma or due to no apparent trauma, with an associated hemorrhage (radiographic diagnosis, hemoglobinemia or hemorrhagic instability), were included. All interventions related to the diagnosis, treatment or prevention of hemorrhage in older patients with pelvic ring fractures were eligible for inclusion. High-energy trauma, fractures caused by a malignant disease and pelvic fractures combined with other severe injuries, such as those caused by polytrauma, were excluded. As this complication is considerably rare, only case reports and case studies were included, and the available information was used to map evidence surrounding hemorrhage in pelvic fractures in older adults. Due to the limited amount of information, no outcome was defined a priori, but the information identified was mapped to provide an overview of the existing information.

### 2.2. Data Search and Selection

An electronic search was performed on Medline via PubMed, Cochrane Central, Web of Science and Bibnet via LIVIVO on 4 April 2022 and on 21 May 2024. The search strategy contained the concepts “pelvic fracture” and “hemorrhage” as well as synonyms. It can be found in the Supplementary Material S1—Search strategy.

After the search was conducted, the detected studies were screened for relevance by two authors (LL and AR) independently. The first step of study selection was the screening of titles and abstracts using the eligibility criteria. Only studies published in English or German were eligible. Thereafter, all selected studies were screened full-text by two authors (LL and AR) independently. A third reviewer (CA or AN) resolved any disagreements. Covidence software (https://www.covidence.org/, accessed on 6 October 2024) was used for screening [9].

### 2.3. Quality Assessment

Due to the nature of the included study designs, no formal risk of bias assessment could be conducted. However, we used the CARE checklist (for CAse REports) to evaluate the reporting of the included studies. The checklist focuses on patients, demographic characteristics, clinical conditions, diagnostic tests, intervention, post-intervention clinical condition, adverse events and takeaway lessons [10]. The checklist was performed for every study that matched the eligibility criteria by two authors (LL and AR) independently. In case of disagreement, a third author (AN) intervened.

### 2.4. Data Extraction

Two authors (LL and AR) independently performed the data extraction after the development and piloting of the data extraction form.

The extracted data included general information (e.g., author, country of origins, journal, year, funding source and conflict of interest), study characteristics (such as study design), patients’ characteristics (age, gender, any family history or lifestyle information provided, disease characteristics (fracture site, affected vessels, anticoagulants, symptoms of shock and general outcome of the hemorrhage) and comorbidities), diagnostic techniques and procedures, therapeutic procedures and patient outcome assessment. Since the systematic review only included case reports or case series, not all the mentioned characteristics were described in every study [11]. A third author (CA or AN) solved any disagreements not resolved via discussion.

### 2.5. Data Synthesis

Due to the nature of the included studies, no meta-analysis was performed. Data were clustered and mapped to be analyzed narratively. Results are presented in tables and illustrations. The goal was to provide an overview of the existing evidence of hemorrhage associated with pelvis ring fracture after low-energy trauma in older adults.

## 3. Results

**Figure 1:**
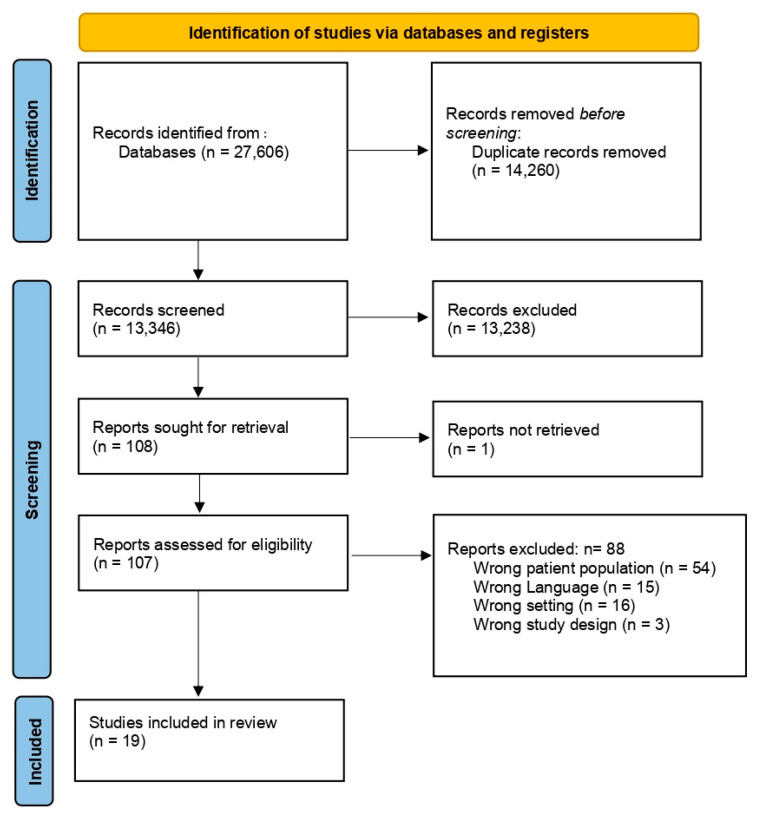
PRISMA flowchart [8].

The search strategy revealed a total of 27,606 hits. After deduplication and title/abstract screening, 108 hits were screened in full text. In total, 19 studies met the inclusion criteria [12,13,14,15,16,17,18,19,20,21,22,23,24,25,26,27,28,29,30] as illustrated in the PRISMA flowchart (Figure 1).

These case reports and case series included a total of 21 patients. A list of excluded studies can be found in the Supplementary Material S2—Excluded studies. Included studies were published between 1988 and 2024; Wingstrand 1988 is the only case reported that was published prior to 2010 [29]. An overview of the study characteristics is provided in Table 2. The studies came from various continents: 12 from Europe, 3 from Asia, 3 from North America and 1 from Australia.

The studies include 17 female and 4 male patients with an average age of 82.1 years (range: 70–96 years; mean female age: 82.4 years; and male mean age: 81 years). All patients had simple falls as an injury mechanism (one study did not report the injury mechanism [20]). A total of 14 studies reported anticoagulant and antiplatelet therapy; 71.4% of patients from these studies received anticoagulants or antiplatelets prior to fracture. One patient suffered an associated compartment syndrome of the thigh [13] and another patient sustained an abdominal compartment syndrome [17]. Three patients (14.3%) had a proximal femur fracture in the past; two of them had received endoprostheses due to this fracture [17,21,25].

**Figure 2:**
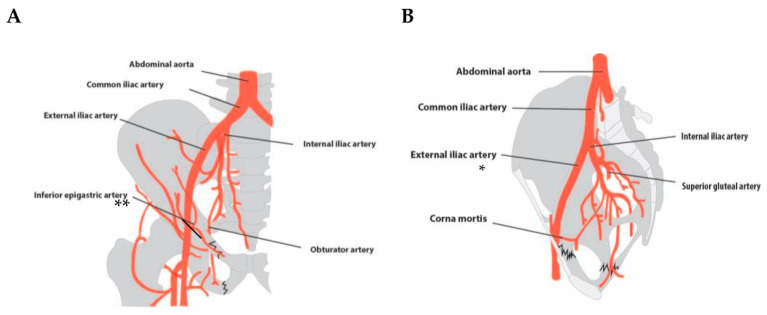
Schematic representation of described injured vessels in ramus pubis fractures: (**A**) coronal perspective; (**B**) sagittal perspective. * The course of the superior gluteal artery is not fully captured in this plane, as it supplies the piriformis muscle and ascends cranially along the dorsal side of the pelvis. Illustration by Ernesto J. Menchaca. ** Pubic branches of the inferior epigastric artery.

**Figure 3:**
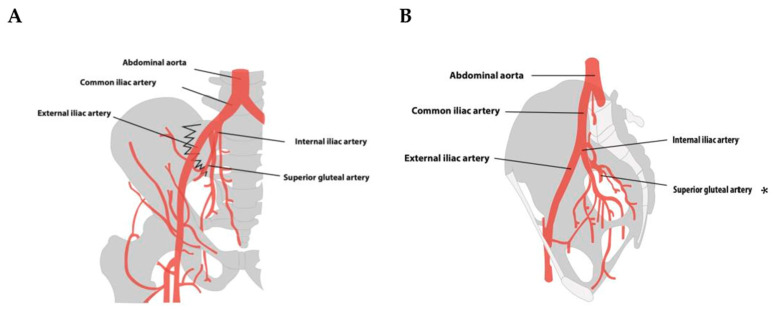
Schematic representation of described injured vessels in sacroiliac fractures: (**A**) coronal perspective; (**B**) sagittal perspective. A sacroiliac fracture cannot be displayed in this plane. * The course of the superior gluteal artery is not fully captured in this plane, as it supplies the piriformis muscle and ascends cranially along the dorsal side of the pelvis. Illustration by Ernesto J. Menchaca.

The 21 patients suffered a total of 29 fractures. Eleven patients had multiple pelvic fractures. The most common fractures were pubic ramus fractures, which were present in 48.3% of all fractures, followed by sacral fractures (13.8% of fractures). Both displaced and non-displaced fractures have been reported in the included studies. A total of 38.1% of patients had at least one (minimally) displaced fracture. More than half of patients (66.7%) suffered from an arterial hemorrhage. In 42.9%, an active hemorrhage was reported. A detailed description of fracture and hemorrhage is shown in Table 3. Arterial vessels were injured in direct anatomical relationship to the fracture. Figure 2 illustrates pubic ramus fractures where the obturator artery and the ramus pubis of the inferior epigastric artery are in close proximity to the fracture site. Figure 3 depicts the anatomical relationship of the superior gluteal artery in sacroiliac fractures. Supplementary Material S3 gives a detailed overview of fractures as described in the studies.

The amount of reported laboratory values was limited. In total, 76.2% of the studies reported at least the hemoglobin level. However, five studies did not report any laboratory results. Beyond that, several studies reported laboratory results of hematocrit, INR, creatinine and lactate [12,17,19,20,21,22,24,26,27].

### 3.1. Symptoms

The leading symptom in all but one study was pain; this study did not report any pain [18]. Patients in studies that reported on pubic ramus fractures had mostly hip or groin pain as the leading symptom. In three of these patients, a later onset of abdominal pain was reported (24 to 72 h after injury) [15,22,24]. Another two patients reported abdominal pain directly at admission examination [12,28]. In patients with sacroiliac fracture, pain was either in the hip, groin or buttock [16,20]. Sixteen patients were found to be hemodynamically unstable during the first 24–72 h after injury. One patient was reported to be hemodynamically unstable in the emergency room [28], and one was considered stable during the course of the hospital stay [16]. Two studies did not report the hemodynamic situation of the patients [20,29]. Studies only reported changes in blood pressure for 14 of the 16 patients with hemodynamic instability. In seven patients, the systolic blood pressure dropped below 100 mmHg, and in seven other patients it dropped below 80 mmHg.

### 3.2. Diagnostics and Therapy

All patients underwent several diagnostic procedures, mostly in the form of imaging. In total, 90.5% of the patients received a CT scan of either the abdomen or only the pelvis, with or without contrast agent. A total of 62% received X-rays of the pelvis, most of them prior to CT imaging. Twelve patients received ultrasound imaging, or the hematoma was monitored with ultrasound. Angiography was performed in 57.1% of patients. Single patients also received an MRI (n = 1) and scintigraphy (n = 2).

The hemorrhage of most patients was treated with embolization (n = 18). Additionally, four patients received surgery as an additional treatment or as the only one. In three patients, no interventional therapy was performed, and they were treated conservatively with blood transfusions and monitoring. One patient died before she could receive any intervention [14].

Additionally, most of the patients received either blood products, clotting factors and/or crystalloid infusions. Fifteen patients received packed red blood cells (PRBCs), with an average of 4.5 units of PRBCs per person (range: 1–10 units). Nine of them also received fresh-frozen plasma (FFP). One patient received FFP and no PRBCs [22]. The average use of FFP was 2.8 units (range: 1–6 units). Additionally, three patients received clotting factors and four received platelets. Only one patient was reported to have received tranexamic acid [23]. Three patients did not receive any blood products or clotting factors [16,17,20] and two studies did not report on it at all [13,29].

A total of 19% of patients (n = 4) died during their hospital stay [14,17,21,23], and for one patient, the outcome is not clear from the case report [18].

### 3.3. Assessment of Reporting

All studies reported at least 50% of the items from the CARE checklist; however, only six studies reported at least 75% of the items. Several items on the CARE checklist were reported in less than 50% of studies, including information on “Medical, family, and psycho-social history with the relevant genetic information” or information regarding the informed consent of patients. The domains patients’ perspective (0%), prognosis (5.3%) and keywords (5.3%) were the least reported items of the checklist, the latter due to missing keywords such as “case report”, as well as the actual diagnosis. A detailed overview of the assessment can be found in the Supplementary Material (S4—Overview of CARE checklist rating).

## 4. Discussion

This systematic review was able to provide an overview of the diagnostic, treatment and preventive options for older patients with pelvic ring fractures. Most evidence retrieved was on patients who suffered pubic ramus fractures. Sparse evidence was found on acetabular fractures. This systematic review extends the findings by Dietz and colleagues (2014) [4], as it includes thirteen more studies and shows that this complication is of increasing concern to the medical community. Hence, a clinical decision tree that summarizes the evidence surrounding diagnostic, treatment and preventive options for ramus pubic fractures was created to serve as an overview of the existing evidence.

**Figure 4:**
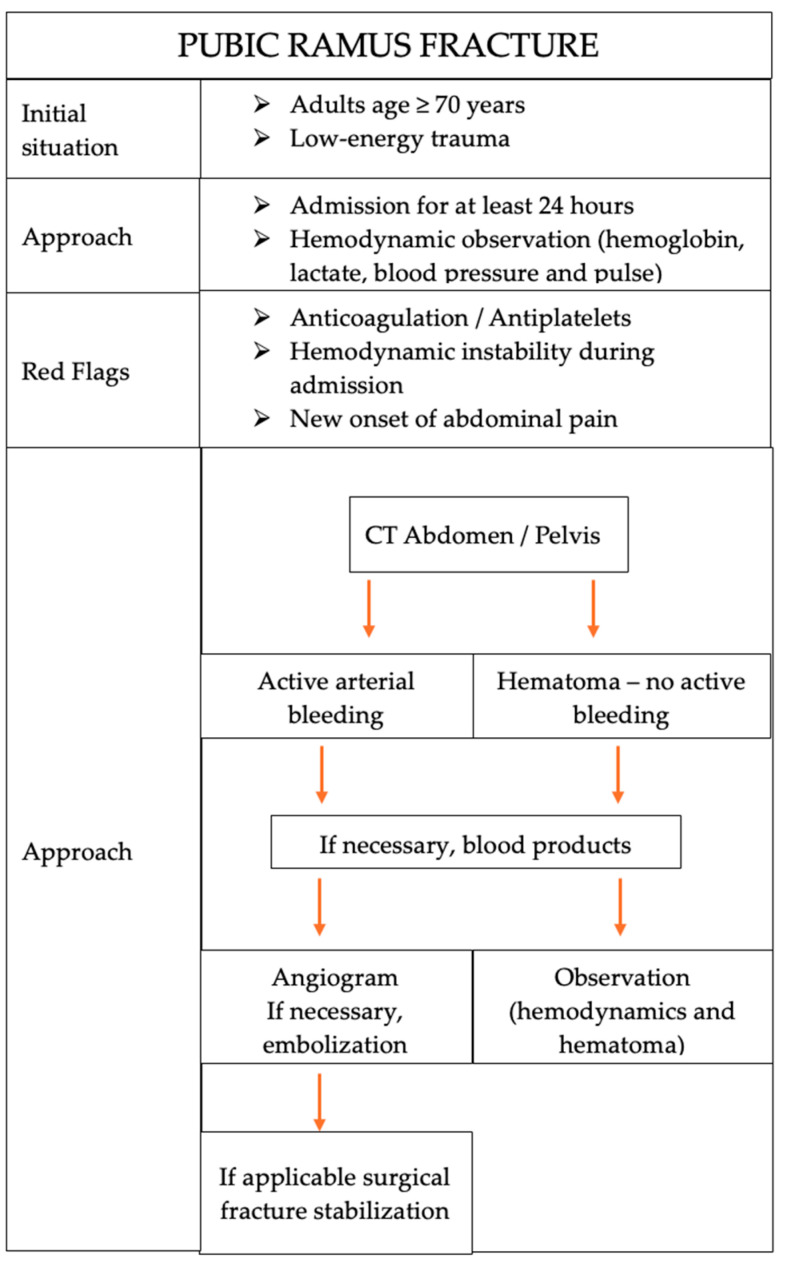
Clinical decision tree for hemorrhage in ramus pubic fractures.

Clinically relevant hemorrhage in pelvic fractures due to low-energy trauma is rare (1, 2, 3). Krappinger and colleagues showed that the relative frequency of severe hemorrhage in elderly patients (>65 years) with pelvic ring fractures could be 2.4%. They believe that this complication will increase in the future due to demographic changes [31]. Studies have shown that these fractures combined with clinically relevant hemorrhage account for an increased mortality and morbidity in elderly people [4,31,32]. Henry and colleagues (2002) showed that older adults with pelvic fractures are more likely to die than younger patients, even after adjusting for their injury severity score [33]. Therefore, we investigated case reports and series to map common symptoms, diagnostics, treatment and prevention strategies, which are summarized in Figure 4. The figure focuses on pubic ramus fractures, as it was only possible to identify patterns for these fractures.

### 4.1. Clinical Implications

Several physiological factors can contribute to a severe hemorrhage after a low-energy trauma pubic ramus fracture in adults older than 55 years, including atherosclerosis, weak connective tissue, a reduction in cardiovascular reserve, as well as anticoagulants. Atherosclerosis may reduce the vessels’ ability for vasospasm and, hence, to self-tamponade the injured vessel, and it makes the arterial wall more fragile. Hence, Krappinger and colleagues believe that elderly patients with atherosclerosis can suffer severe hemorrhage even with stable pelvic ring fractures [31]. Further, age-related physiological changes might weaken connective tissue and cause more fragile vessels to rupture more easily [24,25,33,34].

The results show that the hospital admission of patients with ramus pubic fracture for at least 24 h, with monitoring of their hemodynamic status through blood work (hemoglobin and lactate) in combination with blood pressure and pulse monitoring, as advocated by others, should be carried out [4,14,15,25,35]. Most of the included patients were admitted hemodynamically stable and developed an instability 6 to 72 h after injury due to hemorrhage with visible blood pressure and hemoglobin drop. Additionally, Martin and Casey (2010) pointed out that the reduction in blood pressure should be regarded in relation to the patient’s usual blood pressure range and not in relation to a normative blood pressure [22]. However, due to cardiovascular comorbidities in this age cohort, the definition of hemodynamic instability is challenging, as many suffer high blood pressure or cardiac insufficiency and/or use β-blockers [34].

### 4.2. Red Flags

Patients on anticoagulants and antiplatelets require particular attention due to elevated hemorrhage risks. Several papers highlight the risk of hemorrhage in patients undergoing anticoagulant therapy [4,14,15,18,19,32] and advocate for the admission and monitoring of these patients. We included anticoagulants as a red flag, as particular attention should be paid to such patients. A low threshold for diagnostic imaging should be maintained, especially for patients treated with anticoagulants [31,33,36].

Additionally, several of the included papers highlight the sudden onset of abdominal pain in patients with a diagnosed pubic ramus fracture as a red flag and defined it as an urgent sign of pelvic hemorrhage in some of the patients. As Sandri and colleagues (2014) discuss in their publication, abdominal pain is a wide-ranging symptom in the elderly population [24]. Chang and colleagues add that about 40% of those with abdominal pain are misdiagnosed [37]. Hence, Dietz and colleagues (2014) recommend in their systematic review that two hourly clinical examinations should be performed, including the abdomen, for signs of distention, tenderness, suprapubic swelling or bruising around the groin [4].

### 4.3. Diagnostic and Treatment Approaches

Additionally, we included the two approaches for diagnostics and treatments in Figure 4 to highlight the differential treatments of active hemorrhage and non-active hematoma treatment. The latter refers to the conservatively treated patients, which includes mainly monitoring, rest and pain-related mobilization. CT scans and angiograms were performed as necessary, but embolization was the method of choice in nearly all studies that reported active arterial hemorrhage. Henry and colleagues (2002) point out that many older patients show a delay in obvious bleeding signs due to cardiac dysfunction. They advocate for prudent, early transfusion and early deliberation of the use of angioembolization [33]. Additionally, if necessary, surgery should be performed for, e.g., fracture stabilization. However, the patients included had mostly stable fractures that did not require fracture stabilization. This procedure was also recommended by others [4,32,38]. Further insights into the surgical treatments are beyond the scope of this paper.

### 4.4. Limitations

In this study, a wide search for case reports and case series that fit our inclusion criteria was performed. In comparison to the systematic review by Dietz and colleagues [4] it was possible to identify 13 new case reports. Also, Dietz and colleagues had slightly different inclusion criteria, as Dietz et al. included patients who suffered additional injuries besides the pelvic fracture. Therefore, only six of their eight included studies met our inclusion criteria. There is an increase in literature, especially after 2010, as 13 of the 19 studies included were published after 2010.

It was only possible to identify patterns in red flags, diagnostics and treatments for ramus pubic fractures. The evidence of hemorrhage in acetabular and sacroiliac fractures was not enough to identify patterns.

Evidence is only based on case reports and series and is, hence, limited. However, the study design choice made it possible to map the symptoms, diagnostics and treatments of these rare complications. Hemodynamic instability was not defined prior to this study. Hence, it could be possible that there are differences in when patients were declared hemodynamically unstable in the included studies. Hence, if we could have defined a definition prior to the study, maybe not all patients declared hemodynamically unstable would have met our criteria.

## 5. Conclusions

The rare complication of hemorrhage in older adults with pelvic ring fractures is of growing importance due to demographic changes. This systematic review showed an increase in literature since 2000. The common pattern in symptoms, diagnostics and treatments used allowed the creation of a clinical decision tree, which can serve as an overview of the existing evidence regarding diagnostic, treatment and preventive options for hemorrhage in older adults with pubic ramus fractures. Evidence regarding other fractures, such as acetabulum fractures, is very sparse and did not allow deriving any conclusion for clinical practice. Future studies should focus on analyzing these fractures in multicenter cohort studies (prospectively and retrospectively) to increase the evidence foundation for this complication.

## Figures and Tables

**Table 1:** Inclusion criteria.

	Inclusion Criteria	Exclusion Criteria
Population	Individuals > 55 years Pelvic ring fracture due to a low-energy trauma or due to no apparent trauma and hemorrhage	High-energy trauma Fractures caused by a malignant disease Pelvic fractures that are combined with other severe injuries
Intervention	All interventions related to diagnostics, treatment and/or prevention of hemorrhage in individuals with pelvic ring fractures	
Comparator	No comparator	
Outcome	Not defined	
Study designs	Case reports Case series	Other study designs Systematic reviews Cadaver studies Animal and biomechanical studies

**Table 2:** Characteristics of included studies.

Study ID	Country of Origin	Gender	Age in Years	Mechanism of Injury	Co-Morbidities	Anticoagulant or Antiplatelets Therapy Prior to Fracture	Outcome
Almagauer 2023 [12]	USA	F	86	Ground-level fall	Arterial fibrillation	Eliquis and Plavix	Survived
Burghardt 2010 [13]	Germany	F	94	Fall	Atrial fibrillation Cardiac pacemaker	Marcumar	Survived
Coupe 2005 [14]	UK	F	85	Fall on a patch of ice while walking	Alzheimer’s disease Atrial fibrillation Pernicious anemia	75 mg Aspirin daily	Died
Garrido-Gomez 2012 [15]	Spain	F	70	Fall at home	Osteoporosis	No anticoagulant	Survived
Gómez-Puerta 2008 [16]	Spain	F	83	No apparent trauma	Parkinson’s disease Uterine carcinoma (hysterectomy and radiotherapy 26 years ago)	Ø	Survived
Hagiwara 2004 [17]	Japan	M	84	Fall	Fracture of the neck of the right femur	No anticoagulant	Died
Henning 2007 [18]	Switzerland	F	81	Nonsyncopal fall	Pulmonary embolus 12 years ago Polymyalgia rheumatica Total hysterectomy 21 years ago.	Coumadin derivate	Not reported
Kastanis 2024 [19]	Greece	F	82	Fall from standing height	Arterial hypertension Osteoporosis Hypothyroidism	Acetylsalicylic acid (100 mg once daily)	Survived
Li 2023 [20]	China	F	76	Ø	Ø	Ø	Survived
Macdonald 2006 [21]	UK	F	71	Fall at home	History of deep-venous thrombosis and pulmonary embolism Chronic obstructive airway disease Pulmonary hypertension Osteoporosis Left hip hemiarthroplasty after a fractured neck of the femur	Warfarin	Died
Martin 2010 [22]	Australia	F	89	Fall from standing height	Hypertension Parkinson’s disease Hemorrhoids Recent history of urinary tract infection	No anticoagulant	Survived
Rich 2018 [23]	USA	F	76	Fall from standing height	Ø	Ø	Died
Sandri 2014 [24]	Italy	F	83	Minor fall at home	Chronic atrial fibrillation Osteoporosis	Warfarin	Survived
Solarz 2017 [25]	USA	F	96	Fall from standing height	Hypertension Bilateral proximal femur fractures treated with a right hemiarthroplasty and a left dynamic hip screw	81 mg/d of Aspirin	Survived
ten Broek 2014 [26]	Netherlands	F	79	“Trivial ground-level fall while training with physiotherapist”	Paroxysmal atrial fibrillation Bradycardia	No anticoagulant	Survived
		F	83	“Fall while getting up from the toilet”	Right-sided salpingectomy Chronic diarrhea Hypertension	Ø	Survived
Weber 2016 [27]	Germany	F	78	Ground-level fall	Coronary heart disease Arterial fibrillation Chronic heart failure (NYHA IV) Diabetes Obesity	Coumadin and Acetylsalicylic acid	Survived
Wee 2013 [28]	Singapore	M	73	Simple fall while walking	Stroke	Aspirin 100 mg daily	Survived
Wingstrand 1988 [29]	Sweden	F	81	Fall at home	Ø	Ø	Survived
Wohlrath 2013 [30]	Germany	M	91	Fall on black ice	Ø	Ø	Survived
		F	84	Fall at home	Ø	Ø	Survived

Legend: USA—United States of America.

**Table 3:** Description of fractures and hemorrhage.

Fracture Type	Vessel	Study
Pubic ramus fracture	Internal iliac artery (anterior division)	Henning 2007 [18] Macdonald 2006 [21] Martin 2010 [22] tenBroek 2014 [26]
	Obturator artery (and branches)	Garrido-Gomez 2012 [15] Henning 2007 [18] Rich 2018 [23] Solarz 2017 [25] Wohlrath 2013 [30]
	Superior vesical artery	Wee 2013 [28]
	Corona mortis (connection between obturator artery and inferior epigastric artery)	Henning 2007 [18] Weber 2016 [27] Wohlrath 2013 [30]
	Pubic branches of the inferior epigastric artery	Almagauer 2023 [12] Macdonald 2006 [21] tenBroek 2014 [26] Wohlrath 2013 [30]
Acetabular fracture	Superior gluteal artery	Hagiwara 2004 [17] Wohlrath 2013 [30]
Sacroiliac fracture		Gómez-Puerta 2008 [16]

## Data Availability

The data supporting the conclusions of this article are included within the article and its Supplementary Materials File.

## Supplementary Materials

The following supporting information can be downloaded at: https://www.mdpi.com/article/10.3390/jcm13237223/s1, S1: Search strategy; S2: Excluded studies; S3: Detailed description of fracture; and S4: Overview of CARE checklist rating.
