# Supplementary material for: Hemorrhage in Pelvic Ring Fractures After Low-Energy Trauma: A Systematic Review
Source: J Clin Med. 2024 Nov 28;13(23):7223. doi: 10.3390/jcm13237223 (PMC11642442; doi:10.3390/jcm13237223)
Supplement: Supplementary file 1 [file jcm-13-07223-s001.zip › S1- Search strategy.pdf]

S1 - Search strategy

PubMed

1. ((((fractur*[Title/Abstract])AND (fragilit*[Title/Abstract])) OR (bone fracture[MeSH Terms]))) OR (insufficiency[Title/Abstract])
2. ((((((((((pelvis[Title/Abstract]) OR (pelvic[Title/Abstract])) OR (bone[Title/Abstract])) OR (bone[MeSH Terms])) OR (bone, pelvic[MeSH Terms])) OR (pubic[Title/Abstract])) OR (sacrum[Title/Abstract])) OR (sacral[Title/Abstract])) OR (iliac[Title/Abstract])) OR (pubic rami[Title/Abstract])) OR (bone, pubic[MeSH Terms])) OR (sacrum[MeSH Terms])
3. ((((((((((((hemorrhage[Title/Abstract]) OR (bleeding[Title/Abstract])) OR (bleeder[Title/Abstract])) OR (haemorrhage[Title/Abstract])) OR (hemorrhage[MeSH Terms])) OR (Arteries/injuries[MeSH Terms])) OR (Veins/injuries[MeSH Terms])) OR (Blood Vessels/injuries[MeSH Terms]))) OR ("arterial injury"[Title/Abstract])) OR (venous injury[Title/Abstract])) OR (vessel injury[Title/Abstract])) OR (arter*[Title/Abstract])) OR (vess*[Title/Abstract])

#1 AND #2 AND #3

Cochrane

1. fractur* OR fragilit* OR bone fracture OR insufficiency
2. pelvis OR pelvic OR bone OR (bone AND pelvic) OR pubic OR sacrum OR sacral OR iliac OR pubic rami OR (bone AND pubic)
3. hemorrhage OR bleeding OR bleeder OR haemorrhage OR hemorrhage OR (arteries AND injury) OR (veno* AND injur*) OR (veins* AND injury) OR (blood vessel AND injury) OR arterial injury OR venous injury OR vessel injury OR arter* OR vess

Web of Science

1. fractur* OR fragilit* OR bone fracture OR insufficiency
2. pelvis OR pelvic OR bone OR (bone AND pelvic) OR pubic OR sacrum OR sacral OR iliac OR pubic rami OR (bone AND pubic)
3. hemorrhage OR bleeding OR bleeder OR haemorrhage OR hemorrhage OR (arteries AND injury) OR (veno* AND injur*) OR (veins* AND injury) OR (blood vessel AND injury) OR arterial injury OR venous injury OR vessel injury OR arter* OR vess

bibnet.org via Livivo

1. fractur* OR fragilit* OR bone fracture OR insufficiency
2. pelvis OR pelvic OR bone OR (bone AND pelvic) OR pubic OR sacrum OR sacral OR iliac OR pubic rami OR (bone AND pubic)
3. hemorrhage OR bleeding OR bleeder OR haemorrhage OR hemorrhage OR (arteries AND injury) OR (veno* AND injur*) OR (veins* AND injury) OR (blood vessel AND injury) OR arterial injury OR venous injury OR vessel injury OR arter* OR vess
