# Supplementary material for: Hemorrhage in Pelvic Ring Fractures After Low-Energy Trauma: A Systematic Review
Source: J Clin Med. 2024 Nov 28;13(23):7223. doi: 10.3390/jcm13237223 (PMC11642442; doi:10.3390/jcm13237223)
Supplement: Supplementary file 1 [file jcm-13-07223-s001.zip › S2- Excluded studies.pdf]

S2 – list of excluded studies

Study ID	Reason for exclusion
Abulaban 2011 (1)	Wrong population
Aoki 2020 (2)	Wrong population
Arct 1970 (3)	Language
Babosha 1989 (4)	Language
Balogh 2003 (5)	Wrong setting
Bednarkiewicz 1994 (6)	Language
Benmenachem 1991 (7)	Wrong population
Bölter 2000 (8)	Wrong population
Charney 2000 (9)	Wrong setting
Chaufour 1986 (10)	Language
Chaus 2015 (11)	Wrong population
Chen 2003 (12)	Wrong population
Cheng 1995 (13)	Wrong population
Chiu 2009 (14)	Wrong population
Cho 2021 (15)	Wrong setting
Cormier 1967 (16)	Language
Dietz 2015 (17)	Wrong study design
Dilogo 2021 (18)	Wrong setting
Downs 1988 (19)	Wrong population
Elhence 2020 (20)	Wrong population
Fernández-Lombardía 2014 (21)	Lnguage
Ferrada 2011 (22)	Wrong setting
Gee 2007 (23)	Wrong population
Gerlock 1975 (24)	Wrong population
Ghaemmaghami 2007 (25)	Wrong setting
Graham 2005 (26)	Wrong setting
Grainger 2003 (27)	Wrong population
Haikel 2000 (28)	Wrong population
Hausmann 1975 (29)	Wrong setting
Hessmann 1989 (30)	Wrong population
Hiki 2007 (31)	Wrong population
Horton 1968 (32)	Wrong population
Infanger 1974 (33)	Wrong setting
Johnson-Vaught 2013 (34)	Wrong population
Kawano 2016 (35)	Wrong population
Kimbrell 2004 (36)	Wrong population
Knight 2017 (37)	Wrong setting
Kong 2012 (38)	Wrong setting
Kwon 2023 (39)	Wrong population
Lankford 1999 (40)	Wrong population
Lawson 1968 (41)	Wrong population
Lee 2011 (42)	Wrong population
Likhoded 1963 (43)	Wrong population
Loffroy 2008 (44)	Wrong setting
Lu 2010 (45)	Wrong setting
Mabry 2010 (46)	Wrong population
Mansour 1990 (47)	Wrong population

Marsman 1984 (48)	Wrong population
McHenry 1994 (49)	Wrong population
Meyers 2000 (50)	Wrong population
Mogannam 2017 (51)	Wrong population
Molinero Montes 2021 (52)	Language
Motsay 1972 (53)	Wrong population
Mouzopoulos 2009 (54)	Wrong population
Munihire 2023 (55)	Wrong population
Obón Azuara 2005 (56)	Language
Ozluer 2021 (57)	Wrong population
Palacio 2014 (58)	Language
Parma 1967 (59)	Language
Pascarella 2014 (60)	Wrong population
Patel 1966 (61)	Wrong population
Robin 1968 (62)	Language
Rööser 1989 (63)	Wrong population
Ruotolo 2001 (64)	Wrong population
Sapkas 1968 (65)	Wrong population
Saueracker 1987 (66)	Wrong population
Silberzweig 2009 (67)	Wrong study design
Smith 1976 (68)	Wrong setting
Smith 2005 (69)	Wrong population
Sng 2020 (70)	Wrong population
Songur 2016 (71)	Wrong population
Stein 1981 (72)	Wrong population
Theodorides 2011 (73)	Wrong population
Thompson 2012 (74)	Wrong population
Tosounidis 2010 (75)	Wrong population
Treshev 1964 (76)	Language
Trulson 2018 (77)	Wrong setting
Vichard 1985 (78)	Language
Wang 2023 (79)	Wrong population
Wholey 1998 (80)	Wrong population
Wolinsky 1995 (81)	Wrong population
Xin 2019 (82)	Language
Xu 2018 (83)	Wrong setting
Yang 2021 (84)	Wrong population
Yoon 2004 (85)	Wrong study design
Yosowitz 1972 (86)	Wrong population
Zandi 2022 (87)	Wrong population
Zhang 2005 (88)	Wrong population

References:

1. Abulaban O, Hopkins J, Willis A, Jones R. Pelvic Arterial Embolisation in a Trauma Patient with a Pre-Existing Aortobifemoral Graft. Cardiovasc Intervent Radiol. 2011;34:S102–5.

2. Aoki M, Tokue H, Yajima H, Isshiki Y, Sawada Y, Fukushima K, et al. Selective angioembolization in a pelvic fracture patient with refractory bleeding and hemodynamic instability. 2020; Available from: https://www.livivo.de/doc/M32256923

3. Arct W, Wolny A. [A case of hemorrhagic shock in closed fracture of the pelvic bones]. Wiad Lek. 1970;23(6):473–5.

4. Babosha VA, Zhukov IuB, Lobanov GV, Pasternak VN. [Pulsating intrapelvic hematoma as a complication of a trans-acetabular fracture]. Klin Khir. 1989;(12):53–4.

5. Balogh Z, Vörös E, Süveges G, Simonka JA. Stent graft treatment of an external iliac artery injury associated with pelvic fracture. A case report. J Bone Jt Surg Am. 2003;85(5):919–22.

6. BEDNARKIEWICZ M, PRETRE R, MAURICE J, BRUSCHWEILER I, FAIDUTTI B. SUBADVENTIAL RUPTURE OF THE EXTERNAL ILIAC ARTERY IN BLUNT PELVIC TRAUMA - REPORT OF 2 CASES. Ann Chir. 1994;48(9):850–1.

7. BENMENACHEM Y, COLDWELL D, YOUNG J, BURGESS A. HEMORRHAGE ASSOCIATED WITH PELVIC FRACTURES - CAUSES, DIAGNOSIS, AND EMERGENT MANAGEMENT. Am J Roentgenol. 1991;157(5):1005–14.

8. Bölter S, Haueisen H, Renner N, Roeren T. A. epigastrica inferior. Atypische Blutungsquelle bei Beckenfraktur. 2000; Available from: https://www.livivo.de/doc/M10851959

9. Charny CK, Stanziale SF, Khilnani NM, Helfet DL, Eachempati SR, Barie PS. Unstable pelvic fracture and massive retroperitoneal hematoma from transection of the superior gluteal artery. J Trauma. 2000;48(2):359.

10. Chaufour J, Melki JP, Riche MC, Fernand M, Cormier JM, Laurian C, et al. [Hemorrhagic vascular complications of pelvic fractures . The role of embolization. 9 cases]. Presse Med. 1986;15(42):2097–100.

11. Chaus G, Heng M, Smith R. Occult internal iliac arterial injury identified during open reduction internal fixation of an acetabular fracture: A report of two cases. Inj-Int J CARE Inj. 2015;46(7):1417–22.

12. Chen AL, Wolinsky PR, Tejwani NC. Hypogastric artery disruption associated with acetabular fracture. A report of two cases. J Bone Jt Surg Am. 2003;85(2):333–8.

13. Cheng SL, Rosati C, Waddell JP. Fatal hemorrhage caused by vascular injury associated with an acetabular fracture. J Trauma. 1995;38(2):208–9.

14. Chiu Y, Wong TC, Yeung SH. Haemodynamic instability secondary to minimally displaced pubic rami fractures: a report of two cases. J Orthop Surg Hong Kong. 2009;17(1):100–2.

15. Cho H, Kim Y, Lee J, Yi K, Choi C. Use of N-butyl cyanoacrylate in the successful transcatheter arterial embolization of an arteriovenous fistula caused by blunt pelvic fracture A case report and review of literature. Medicine (Baltimore). 2021;100(1).

16. Cormier JM, Florent J. [Retroperitoneal hematoma due to injury of the iliac vessels associated with pelvic fractures]. Mem Acad Chir Paris. 1967;93(15):458–63.

17. Dietz SO, Hofmann A, Rommens PM. Haemorrhage in fragility fractures of the pelvis. Eur J Trauma Emerg Surg [Internet]. 2015 Aug [cited 2024 Sep 18];41(4):363–7. Available from: http://link.springer.com/10.1007/s00068-014-0452-1

18. Dilogo I, Prabowo I. The role of angioembolization and C-clamp fixation: Damaged control orthopaedic in haemodynamically unstable pelvic fracture. Ann Med Surg. 2021;63.

19. Downs AR, Dhalla S. Hemorrhage and pelvic fractures. Can J Surg. 1988;31(2):89–90.

20. Elhence A, Gahlot N, Gupta A, Garg P. Internal Pudendal Artery Injury Following An Open Book Pelvic Fracture. 2020; Available from: https://www.livivo.de/doc/M33403083

21. Fernández-Lombardía J, Paz-Aparicio A, Hernández-Vaquero D. [Vascular complications after pelvic rami fracture]. Rev Esp Cir Ortop Traumatol. 2014;58(6):407–10.

22. Ferrada PA, Jain K. Aberrant obturator artery: a hidden threat after pelvic fracture. Am Surg. 2011;77(6):e126.

23. Gee M, Tolat A, Sinha J. Acute gluteal and thigh compartment syndrome following pelvic fracture and superior gluteal artery bleed - A case report and review of the literature. Eur J TRAUMA Emerg Surg. 2007;33(2):188–91.

24. Gerlock AJ. Hemorrhage following pelvic fracture controlled by embolization: case report. J Trauma. 1975;15(8):740–2.

25. Ghaemmaghami V, Sperry J, Gunst M, Friese R, Starr A, Frankel H, et al. Effects of early use of external pelvic compression on transfusion requirements and mortality in pelvic fractures. Am J Surg. 2007;194(6):720–3.

26. Graham CP. Fatal haemorrhage following a low-energy fracture of the pubic ramus. J Bone Jt Surg Br. 2005;87(12):1700.

27. Grainger MF, Porter KM. Life threatening haemorrhage from obturator vessel tear as a result of pubic ramus fracture. Injury. 2003;34(7):543–4.

28. Haikel S, Willett K. Traumatic rupture of the superior gluteal artery with a stable pelvic fracture. Injury. 2000;31(5):383–6.

29. Hausmann E. Zur Kasuistik eines Falles mit Beckenbruch und schwerer Gefässverletzung. 1975; Available from: https://www.livivo.de/doc/M1205946

30. Hessmann M, Rommens P. Bilateral ureteral obstruction and renal failure caused by massive retroperitoneal hematoma: Is there a pelvic compartment syndrome analogous to abdominal compartment syndrome? J Orthop TRAUMA. 1998;12(8):553–7.

31. Hiki T, Okada Y, Wake K, Fujiwara A, Kaji Y. Embolization for a bleeding pelvic fracture in a patient with persistent sciatic artery. Emerg Radiol. 2007;14(1):55–7.

32. Horton RE, Hamilton GI. Ligature of the internal iliac artery for massive haemorrhage complicating fracture of the pelvis. J Bone Joint Surg Br. 1968;50(2):376–9.

33. Infanger K, Gianella F, Schamaun M. [Ligation of the internal iliac artery in pelvic fractures with massive uncontrollable hemorrhage]. Helv Chir Acta. 1974;41(1–2):247–9.

34. Johnson-Vaught LD. Comminuted pelvic fracture with retroperitoneal bleed in a geriatric patient. 2013; Available from: https://www.livivo.de/doc/M23797615

35. Kawano T, Miyakoshi N, Suzuki T, Kasukawa Y, Ishikawa N, Tazawa H, et al. Chronic expanding hematoma resulting from osteoporotic insufficiency fracture of the pubis. J Orthop Sci. 2016;21(6):865–9.

36. Kimbrell B, Velmahos G, Chan L, Demetriades D. Angiographic embolization for pelvic fractures in older patients. Arch Surg. 2004;139(7):728–33.

37. Knight C, Mboumi I, Thompson E. Severe pelvic fracture with profound hypotension: a case report and treatment algorithm. J Surg CASE Rep. 2017;(6).

38. Kong D, Fan X, Song C, Wu M, Wu L, Yang T, et al. A Comparative Analysis of Between Percutaneous Cannulated Screw Fixation and Traditional Plate Internal Fixation in Treatment of Sanders II and III Calcaneal Fractures. [Internet]. 2023. Available from: https://www.livivo.de/doc/M38151111

39. Kwon H, Jang JH, Moon NH, Rhee SJ, Ryu DY, Ahn TY. Superior gluteal artery injury in pelvic ring injury and acetabular fracture. 2023; Available from: https://www.livivo.de/doc/M37867061

40. Lankford A, Senkowski CK. Bilateral external iliac artery dissections after pelvic fracture: case report. J Trauma. 1999;47(4):784–6.

41. Lawson LJ, Wainwright D. Massive haemorrhage following pelvic fracture. Report of a case. J Bone Joint Surg Br. 1968;50(2):380–2.

42. Lee M, Haene RA, Fonseka S, Khanduja V. Superior gluteal artery rupture associated with an isolated fracture of the sacrum. Injury. 2011;42(7):719–21.

43. LIKHODED SI, FERUZ AS. [ON RETROPERITONEAL INTERSTITIAL HEMORRHAGE IN PELVIC BONE FRACTURES AND COMPLEX CENTRAL HIP DISLOCATIONS]. Ortop Travmatol Protez. 1963;24:19–22.

44. Loffroy R, Yeguiayan J, Guiu B, Cercueil J, Krause D. Stable fracture of the pubic rami: a rare cause of life-threatening bleeding from the inferior epigastric artery managed with transcatheter embolization. Can J Emerg Med. 2008;10(4):392–5.

45. Lu C, Lee Y, Sun P, Liang C, Liliang P. Life-threatening bleeding from the pubic branch of the inferior epigastric artery after pubic ramus fracture. HONG KONG J Emerg Med. 2010;17(4):372–6.

46. Mabry LM, Ross MD, Tall MA. Insufficiency fracture of the pubic rami. J Orthop Sports Phys Ther. 2010;40(10):666.

47. Mansour MA, Moore FA, Moore EE. Hypogastric arterial embolization in pelvic fracture hemorrhage: case report. J Trauma. 1990;30(11):1417–8.

48. Marsman JW, Schilstra SH, van Leeuwen H. Angiography and embolization of the corona mortis (aberrant obturator artery). A source of persistent pelvic bleeding. Rofo. 1984;141(6):708–10.

49. McHenry CR, Jacobs DG. Pelvic hematoma necessitans--a delayed complication of massive hemorrhagic pelvic fracture. 1994; Available from: https://www.livivo.de/doc/M8015015

50. Meyers TJ, Smith WR, Ferrari JD, Morgan SJ, Franciose RJ, Echeverri JA. Avulsion of the pubic branch of the inferior epigastric artery: a cause of hemodynamic instability in minimally displaced fractures of the pubic rami. J Trauma. 2000;49(4):750–3.

51. Mogannam A, Cubas R, Gutierrez I, Astudillo J, Abou-Zamzam A. Blunt Traumatic Occlusion of the Common Iliac Artery Repaired With Segmental Excision and Internal Iliac Artery Patch Angioplasty. Ann Vasc Surg. 2017;39.

52. Molinero Montes M, Fernández Álvarez C, Fernández-Valdés Fernández JM. Study of hemodynamic instability due to intrapelvic hemorrhage as a consequence of ilioischiopubian branch fractures in geriatric patients. Rev Espanola Cirugia Ortop Traumatol. 2021;

53. Motsay GJ, Alho A, Butler B, Perry JF, Lillehei RC. Iliac vein trauma with pelvic fracture. Postgrad Med. 1972;51(2):133–6.

54. Mouzopoulos G, Tzurbakis M, Mouzopoulos D, Ierodiakonou V, Tsembeli A, Georgilas I. Massive haemorrhage due to minimally displaced pubic ramus fracture. Eur J Emerg Med. 2009;16(5):271–2.

55. Munihire JB, Balarabe HS, Olasinde AA, Muhumuza J. Complex pelvic fracture with massive hemorrhage in low resource settings. 2023; Available from: https://www.base-search.net/Search/Results?lookfor=baseid:ftdoajarticles:oai:doaj.org/article:2782857f721d4a18b61a52e8315329fb

56. Obón Azuara B, Villanueva Anadón B, Gutiérrez Cía I, Cárcamo Merino A, Montoiro Allué R, Martín Villel L. [Peritoneal hematoma due to bleeding in patient with pelvic fracture]. Med Interna. 2005;22(12):605–6.

57. Ozluer Y, Avcil M, Dizman S. Successful angioembolization with autologous subcutaneous fat in an open book pelvic fracture. Turk J Emerg Med. 2021;21(2):79–81.

58. Palacio J, Albareda J. [Severe haemorrhage secondary to an osteoporotic pelvic fracture: presentation of a case]. Rev Esp Cir Ortop Traumatol. 2014;58(3):192–5.

59. Parma A. [Large pelvic hematoma secondary to fracture of the pelvis. (Pathogenetic and clinical considerations)]. Acta Chir Ital. 1967;23(1):47–53.

60. Pascarella R, Del Torto M, Politano R, Commessatti M, Fantasia R, Maresca A. Critical review of pelvic fractures associated with external iliac artery lesion: A series of six cases. Inj-Int J CARE Inj. 2014;45(2):374–8.

61. Patel NH, Matsuo RT, Routt ML Jr. An acetabular fracture with superior gluteal artery disruption. AJR Am J Roentgenol. 1996;166(5):1074.

62. Robin B, Bonnaud P. [Hemorrhagic complications of fractures of the pelvis]. J Chir Paris. 1968;96(3):189–92.

63. Rööser B, Bengtson S, Herrlin K. A case of acetabular fracture with hip tamponade. Relief from aspiration of hemarthrosis. Acta Orthop Scand. 1989;60(5):623–4.

64. Ruotolo C, Savarese E, Khan A, Ryan M, Kottmeier S, Meinhard BP. Acetabular fractures with associated vascular injury: a report of two cases. J Trauma. 2001;51(2):382–6.

65. Sapkas A, Tierris E. [Ligation of the arteria iliaca interna in uncontrolable bleeding following severe pelvic fractures]. Langenbecks Arch Chir. 1968;322:1102–8.

66. Saueracker AJ, McCroskey BL, Moore EE, Moore FA. Intraoperative hypogastric artery embolization for life-threatening pelvic hemorrhage: a preliminary report. J Trauma. 1987;27(10):1127–9.

67. Silberzweig JE. Re: Transcatheter arterial embolization for pelvic fractures may potentially cause a triad of sequela: gluteal necrosis, rectal necrosis, and lower limb paresis. J Trauma. 2009;67(2):416–7.

68. Smith K, Ben-Menachem Y, Duke JH Jr, Hill GL. The superior gluteal: an artery at risk in blunt pelvic trauma. J Trauma. 1976;16(4):273–9.

69. Smith WR, Moore EE, Osborn P, Agudelo JF, Morgan SJ, Parekh AA, et al. Retroperitoneal packing as a resuscitation technique for hemodynamically unstable patients with pelvic fractures: report of two representative cases and a description of technique. J Trauma. 2005;59(6):1510–4.

70. Sng M, Gentle J, Asadollahi S. Bleeding Risk Associated With Hemodynamically Stable Low-Energy Pelvic Fracture. Geriatr Orthop Surg Rehabil. 2020;11.

71. Songur M, Şahin E, Zehir S, Oz II, Kalem M. Gluteal compartment syndrome secondary to superior gluteal artery injury following pelvis fracture. 2016; Available from: https://www.livivo.de/doc/M27239636

72. Stein BR, Kerber CW. Therapeutic arterial embolization for posttraumatic hemorrhage: report of case. J Oral Surg. 1981;39(6):439–41.

73. Theodorides AA, Morgan BW, Simmons D. Haemodynamic instability resulting from a low energy pubic ramus fracture in a 78-year-old woman. A case report and review of the literature. Injury. 2011;42(7):722–4.

74. Thompson SM, Giles MF. A complicated pelvic fracture in an octogenarian. QJM. 2012;105(1):81–2.

75. Tosounidis G, Culemann U, Stengel D, Garcia P, Kurowski R, Holstein J, et al. Complex pelvic trauma in elderly patients. UNFALLCHIRURG. 2010;113(4):281–6.

76. TRESHEV VS, MATYSHEV AA. [THE COURSE OF SPREADING OF HEMATOMA FOLLOWING CLOSED FRACTURES OF THE PELVIS]. Vestn Khir Im Grek. 1964;93:59–64.

77. Trulson A, Kuper M, Trulson I, Minarski C, Stockle U, Stuby F. Fracture of the anterior pelvic ring-the alleged minor injury. TRAUMA Berufskrankh. 2018;20:157–62.

78. Vichard P, Zeil A. [Severe subperitoneal hemorrhage in fractures of the pelvis]. Chirurgie. 1985;111(3):229–34.

79. Wang M, Zhao R, Hao Y, Xu P, Lu C. Return to work status of patients under 65 years of age with osteonecrosis of the femoral head after total hip arthroplasty. [Internet]. 2023. Available from: https://www.livivo.de/doc/M37853426

80. Wholey M, Peterson S, Silvestri B. Case 2: Pelvic fracture with tear of the left internal pudendal artery. AJR Am J Roentgenol. 1998;171(3):844, 847, 848.

81. Wolinsky PR, Johnson KD. Delayed catastrophic rupture of the external iliac artery after an acetabular fracture. A case report. J Bone Joint Surg Am. 1995;77(8):1241–4.

82. Xin HL, Liang JB, Yang Y, Chen ZY, Hong HX, Zhang Q. [Corona Mortis artery injury caused by simple pubic ramus fracture:a case report]. Zhongguo Gu Shang. 2019;32(7):674–6.

83. Xu X, Teng F, Li J, Wu J, Zhu R, Ji W. Life-threatening hemorrhage from the corona mortis treated with balloon-assisted coiling technique. Am J Emerg Med. 2018;36(3).

84. Yang Z, Meng X, Fu P. Treatment for Laceration of Arterial Corona Mortis and Huge Retropubic Hematoma in an Elderly Man. INDIAN J Surg. 2021;83(SUPPL 1):S243–6.

85. Yoon W, Kim JK, Jeong YY, Seo JJ, Park JG, Kang HK. Pelvic arterial hemorrhage in patients with pelvic fractures. 2004; Available from: https://www.livivo.de/doc/M15537967

86. Yosowitz P, Hobson RW 2nd, Rich NM. Iliac vein laceration caused by blunt trauma to the pelvis. Am J Surg. 1972;124(1):91–3.

87. Zandi R, Hassani M, Manafi A, Meibodi MKE. External Iliac Artery Injury Occurring After Pelvic Ring and Acetabulum Fracture: A Rare Case Report. Trauma Mon. 2022;27(2):402–6.

88. Zhang FQ, Zhang YZ, Pan JS, Peng AQ, Wang HJ. Pelvic compartment syndrome caused by retroperitoneal hematoma of pelvic fracture. Chin Med J Engl. 2005;118(10):877–8.
