# Supplementary material for: Hemorrhage in Pelvic Ring Fractures After Low-Energy Trauma: A Systematic Review
Source: J Clin Med. 2024 Nov 28;13(23):7223. doi: 10.3390/jcm13237223 (PMC11642442; doi:10.3390/jcm13237223)
Supplement: Supplementary file 1 [file jcm-13-07223-s001.zip › S3- Detailed description of fractures.pdf]

S3 Detailed description of fractures

Study ID	Description of fracture
**Almagauer 2023**	"Mildly comminuted, **nondisplaced fracture of the left pubic body and superior pubic ramus** as well as a **nondisplaced fracture of the right inferior pubic ramus** and **right sacral wing"**
**Burghardt 2010**	"Nondisplaced **anterior pelvic ring fracture** on the **left** side"
**Coupe 2005**	"Isolated, minimally displaced fracture of the **left superior pubic ramus"**
**Garrido-Gomez 2012**	"**Left iliopubic rami fracture** and a nondisplaced **right ischio-pubic rami fracture"**
**Gómez-Puerta 2008**	"Displaced **right sacro-iliac fracture** and **left iliac** and **ischio-pubic ramus** fractures"
**Hagiwara 2004**	"Fractures of the **right pubis** and **ischium** and a **fracture from the region of the right acetabular cartridge to the right iliac bone**"
**Henning 2007**	"**Left** nondisplaced **superior and inferior pubic ramus fractures"**
**Kastanis 2024**	"Displaced **right superior pubic ramus** fracture and a nondisplaced **right inferior pubic ramus fracture**"
**Li 2023**	"Fractures of the **left sacral ala** **and the left pubic bone**"
**Macdonald 2006**	"Minimally displaced, stable fracture of the **left superior pubic ramus"**
**Martin 2010**	"**Right pubic rami fractures** and a **nondisplaced right sacral fracture**"
**Rich 2018**	"Non-displaced fractures through the **right superior and inferior pubic rami**"
**Sandri 2014**	"**Right** osteoporotic **pubic rami fracture** classified as type Ia according to the Rommens-Hofmann classification"
**Solarz 2017**	"Minimally displaces **right superior pubic ramus** fracture"
**tenBroek 2014**	"**Right superior pubic rami** fracture without dislocation"
	"Fracture of the **left superior and inferior pubic rami"**
**Weber 2016**	"Fragility fracture of the pelvis, simple **iliopubic rami fracture right**, benign fracture pattern, osteoporotic **sacral fracture** (Denis type I), partially unstable fragility fracture of the pelvis type IIC"
**Wee 2013**	"Minimally displaced **right superior pubic ramus fracture**; no significant displacement"
**Wingstrand 1988**	"Minor, displaced **acetabular fracture**"
**Wohlrath 2013**	"**Left iliac wing** fracture with **2-pillar acetabular fracture**, simple **anterior pelvic ring fracture** (type A according to AO)"

	"Slightly displaced **anterior pelvic ring** fracture with marginally dislocated **pubic ramus fracture** (type A according to AO)"
