# Supplementary material for: Hemorrhage in Pelvic Ring Fractures After Low-Energy Trauma: A Systematic Review
Source: J Clin Med. 2024 Nov 28;13(23):7223. doi: 10.3390/jcm13237223 (PMC11642442; doi:10.3390/jcm13237223)
Supplement: Supplementary file 1 [file jcm-13-07223-s001.zip › S4- CaRe Checklist rating.pdf]

Supplemental file S4: Care Checklist Items per study

Care Checklist Items			Almagauer 2023	Burghardt 2010	Coupe 2005	Garrido-Gomez 2012	Gómez-Puerta 2008	Hagiwara 2004	Henning 2007	Kastanis 2024	Li 2023	Macdonald 2006	Martin 2010	Rich 2018	Sandri 2014	Solarz 2017	tenBroek 2014	Weber 2016	Wee 2013	Wingstrand 1988	Wohlrath 2013	**Total**	**Percentage of studies that reported this item**
Title	1	The diagnosis or intervention of primary focus followed by the words "case report"	1	1	1	1	0	1	0	1	0	1	1	1	1	1	0	0	1	1	0	12	63%
Key Words	2	2 to 5 key words that identify diagnoses or interventions in this case report, including "case report"	1	0	0	0	0	0	0	0	0	0	0	0	0	0	0	0	1	0	0	1	5.3%
Abstract	3a	Introduction: What is unique about this case and what does it add to the scientific literature?	0	1	0	1	1	1	1	1	1	1	1	1	1	1	1	1	1	1	0	16	84.2%
(no references)	3b	Main symptoms and/or important clinical findings	1	1	1	1	1	1	1	1	0	1	1	1	1	1	1	1	1	1	1	17	89.5%
	3c	The main diagnoses, therapeutic interventions, and outcomes	1	1	0	1	1	1	1	1	1	0	1	1	1	1	1	1	1	1	1	16	84.2%
	3d	Conclusion—What is the main "take-away" lesson(s) from this case?	1	1	0	1	1	1	1	1	1	1	1	1	1	1	1	1	1	1	1	17	89.5%
Introduction	4	One or two paragraphs summarizing why this case is unique (may include references)	1	1	1	1	0	1	1	1	1	1	1	1	1	1	1	1	1	1	1	17	89.5%
Patient Information	5a	De-identified patient specific information	1	1	1	1	1	1	1	1	1	1	1	1	1	1	1	1	1	1	1	18	94.7%
	5b	Primary concerns and symptoms of the patient	1	0	1	1	1	1	0	1	0	1	1	1	1	0	0	0	0	1	1	11	57.9%

	5c	Medical, family, and psycho-social history including relevant genetic information	1	0	0	0	1	0	1	0	0	1	1	1	1	1	0	1	1	0	0	9	47.4%
	5d	Relevant past interventions with outcomes	0	0	0	0	1	0	1	0	0	1	0	0	0	1	0	0	1	0	0	5	26%
Clinical Findings	6	Describe significant physical examination (PE) and important clinical findings	1	1	1	1	1	1	1	1	1	1	1	1	1	1	1	1	1	1	1	18	94.7%
Timeline	7	Historical and current information from this episode of care organized as a timeline	1	1	1	1	1	1	1	1	1	1	1	1	1	1	0	1	1	1	1	17	89.5%
Diagnostic Assessment	8a	Diagnostic testing (such as PE, laboratory testing, imaging, surveys)	1	1	1	1	1	1	1	1	1	1	1	1	1	1	1	1	1	1	1	18	94.7%
	8b	Diagnostic challenges (such as access to testing, financial, or cultural)	1	0	0	0	0	0	1	0	0	0	1	0	0	1	0	0	1	0	0	4	21.1%
	8c	Diagnosis (including other diagnoses considered)	1	1	1	1	1	1	1	1	1	1	1	1	1	1	1	1	1	1	1	18	94.7%
	8d	Prognosis (such as staging in oncology) where applicable	0	0	0	0	0	0	0	0	0	1	0	0	0	0	0	0	0	0	0	1	5.3%
Therapeutic Intervention	9a	Types of therapeutic intervention (such as pharmacologic, surgical, preventive, self-care)	1	1	1	1	1	1	1	1	1	1	1	1	1	1	1	1	1	1	1	18	94.7%
	9b	Administration of therapeutic intervention (such as dosage, strength, duration)	1	1	0	0	0	1	1	1	1	1	1	0	1	1	0	1	1	1	1	13	68.4%
	9c	Changes in therapeutic intervention (with rationale)	1	1	1	1	1	1	1	1	0	1	1	1	1	1	0	1	1	1	1	16	84.2%
Follow-up and Outcomes	10a	Clinician and patient-assessed outcomes (if available)	1	1	1	0	1	0	0	1	0	1	0	1	1	1	0	1	1	1	0	11	57.9%
	10b	Important follow-up diagnostic and other test results	0	1	0	0	0	0	0	1	1	0	1	0	1	1	1	1	0	1	0	9	47.4%

	10c	Intervention adherence and tolerability (How was this assessed?)	0	1	0	0	0	0	0	0	0	1	1	1	0	1	0	1	0	1	0	7	36.8%
	10d	Adverse and unanticipated events	1	1	1	1	1	1	0	0	0	1	1	1	1	1	1	1	1	0	0	13	68.4%
Discussion	11a	A scientific discussion of the strengths AND limitations associated with this case report	0	1	1	1	1	0	0	0	0	1	0	1	0	0	0	0	0	0	0	6	31.6%
	11b	Discussion of the relevant medical literature with references	1	1	1	1	1	1	1	1	1	1	1	1	1	1	1	1	1	0	1	17	89.5%
	11c	The scientific rationale for any conclusions (including assessment of possible causes)	1	1	1	1	1	1	1	1	1	1	1	1	1	1	1	1	1	1	1	18	94.7%
	11d	The primary "take-away" lessons of this case report (without references) in a one paragraph conclusion	0	1	1	1	1	1	0	1	0	1	1	1	1	1	1	1	1	1	1	16	84.2%
Patient Perspective	12	The patient should share their perspective in one to two paragraphs on the treatment(s) they received	0	0	0	0	0	0	0	0	0	0	0	0	0	0	0	0	0	0	0	0	0%
Informed Consent	13	Did the patient give informed consent? Please provide if requested	0	1	0	0	0	0	0	1	1	0	1	0	1	0	0	1	0	0	0	6	31.6%
		Total per study (30 possible points)	21	23	17	19	20	19	18	21	15	24	24	22	23	24	15	22	23	20	16		
		Percentage of sufficiently reported items	70%	76.7%	56.7%	63.3%	66.7%	63.3%	60%	70%	50%	80%	80%	73.3%	76.7%	80%	50%	73.3%	76.7%	66.7%	53.3%		

Legend: item reported; item not reported; ≥ 50% reported items, ≥75% reported items
